# Spirometric reference equations and lung function testing in adults from Southwestern Tanzania

**DOI:** 10.5588/ijtldopen.24.0339

**Published:** 2024-10-01

**Authors:** R. Wenzel, E. Siyame, O. Ivanova, A. Bakuli, J. Lalashowi, F.C. Zekoll, M. Hoelscher, I. Sabi, A. Rachow, N.E. Ntinginya

**Affiliations:** ^1^ Institute of Infectious Diseases and Tropical Medicine, Ludwig Maximilian University (LMU) University Hospital, LMU Munich, Munich, Germany;; ^2^ Mbeya Medical Research Centre, National Institute for Medical Research (NIMR), Mbeya, Tanzania;; ^3^ Assistant Medical Officers Training School, Ministry of Health, Mbeya, Tanzania;; ^4^ German Centre for Infection Research (DZIF), Partner Site Munich, Germany;; ^5^ Unit Global Health, Helmholtz Zentrum München, German Research Center for Environmental Health (HMGU), Neuherberg, Germany.

**Keywords:** LFT, spirometry, prediction equation, adult, Tanzania, Africa

## Abstract

**BACKGROUND:**

The increasing relevance of lung function testing in diagnosing and treating pulmonary diseases globally requires adequate spirometric reference equations. However, locally derived reference standards from African countries are widely missing.

**METHODS:**

This cross-sectional study was conducted in Southwestern Tanzania. Participants underwent a socio-demographic interview, anthropometric measurements and standardised spirometric lung function testing. Regression modelling was used to generate Tanzanian prediction equations for spirometry parameters forced vital capacity (FVC), forced expiratory volume in 1 sec (FEV_1_) and the FEV_1_/FVC ratio.

**RESULTS:**

Out of 400 recruited participants, 343 had usable spirometry results with respect to the American Thoracic Society (ATS)/European Respiratory Society (ERS) reproducibility and acceptability criteria. The mean age of participants was 32.65 years (SD 12.11), and 44,9% were females. Spirometric parameters increased with height but decreased with older age. The coefficients of our new prediction equations for spirometry parameters differed substantially from those of existing reference standards.

**CONCLUSION:**

This study provides prediction equations for spirometric lung function in a non-smoking Tanzanian population. The differences in existing equations underline the heterogeneity of locally derived reference equations in Africa and contribute insights and data to discussing global respiratory health care reference standards.

## RÉSUMÉ

**CONTEXTE:**

L'importance croissante des tests de la fonction pulmonaire pour le diagnostic et le traitement des affections respiratoires à l'échelle mondiale met en lumière la nécessité d'équations spirométriques de référence adéquates. Néanmoins, les normes de référence élaborées localement dans les pays africains sont largement insuffisantes.

**MÉTHODES:**

Cette recherche transversale a été réalisée dans le sud-ouest de la Tanzanie. Les participants ont été soumis à un entretien sociodémographique, à des mesures anthropométriques ainsi qu'à des tests spirométriques standardisés pour évaluer la fonction pulmonaire. La modélisation par régression a permis de développer des équations de prédiction spécifiques à la Tanzanie pour les paramètres spirométriques suivants : capacité vitale forcée (CVF), volume expiratoire forcé en une seconde (VEMS) et le rapport VEMS/CVF.

**RÉSULTATS:**

Parmi les 400 participants recrutés, 343 ont fourni des résultats de spirométrie jugés utilisables selon les critères de reproductibilité et d'acceptabilité établis par l'American Thoracic Society (ATS) et l'European Respiratory Society (ERS). L'âge moyen des participants s'élevait à 32,65 ans (déviation standard : 12.11), avec une proportion de 44,9% de femmes. Les paramètres spirométriques étaient en augmentation avec la taille, tandis qu'ils diminuaient avec l'âge. Les coefficients de nos nouvelles équations de prédiction des paramètres spirométriques se distinguaient de manière significative de ceux des normes de référence actuellement disponibles.

**CONCLUSION:**

Cette étude présente des équations de prédiction de la fonction pulmonaire spirométrique mesurée par spirométrie au sein d'une population tanzanienne non fumeuse. Les variations observées entre les équations déjà établies mettent en évidence la diversité des équations de référence élaborées localement en Afrique et alimentent le débat sur les normes de référence internationales en matière de soins respiratoires.

Spirometry is the most commonly used lung function test available, and it constitutes an important tool for diagnosing and monitoring pulmonary diseases.^1,2^ It measures the volume and flow of exhaled air. Those measurements are depicted in the typical lung function parameters: forced vital capacity (FVC), forced expiratory volume in one second (FEV_1_) and the FEV1/FVC ratio.

To interpret spirometry results, the obtained values for lung function parameters according to the American Thoracic Society/European Respiratory Society (ATS/ERS) recommendations should be compared to reference values, which originate from a population with the same ethnic and anthropometric background.^3^ Ideally, reference values should be obtained from a representative, sufficiently large, healthy and non-smoking population.^3^ Therefore, the Global Lung Function Initiative (GLI) was initiated to establish continuous prediction equations and lower limits of normal (LLN) for spirometric indices applicable globally.^4^ GLI recently published a ‘race-neutral’ reference standard, a composite of the four previously published standards. By removing race and ethnicity from the calculation of predicted lung function, the aim was to address the disparities promoted by current race and ethnicity-specific equations.^5^

Masekela et al. highlighted the need for an African standard,^6^ as only a few African data points are included in GLI. Therefore, our study aims to contribute up-to-date reference equations from Southwestern Tanzania's representative, self-reported, healthy and non-smoking adult population.

## METHODS AND MATERIALS

### Study design and participants

We conducted a cross-sectional study between April and October 2019 in the Mbeya and Songwe Regions (population: 3.69 million).^7^ Most of the population has attended primary school only.^8^ Leading causes of mortality are respiratory infections, HIV/AIDS and malaria.^8^

Our sample size was based on Quanjer et al., who estimated the number of subjects necessary to validate reference values of at least 150 males and 150 females.^9^

Study participants were recruited from the same neighbourhoods as study participants of a TB Sequel cohort study, a study to demonstrate the clinical spectrum of TB-related lung injury.^10^ Respiratory asymptomatic, non-smoking adults were included in the study. Participants were excluded if they had any signs/history of chronic pulmonary disease, especially any current or past TB. Contraindications to perform spirometry included a history of cardiovascular disease, muscular disorders, glaucoma, sickle cell disease, recent chest trauma, surgery or hospitalisation.

### Data collection

Participants were interviewed to check for inclusion criteria and underlying contraindications and to collect socio-demographic data. Body weight and standing height were collected in a standardised manner.

Spirometry was performed using the handheld ndd EasyOne Air (ndd Medizintechnik, Basel, Switzerland), it has already been validated in several previous studies.^11,12^ Spirometry tests were performed according to ATS/ERS guidelines (online supplement to article). An extensive quality control process was implemented to identify usable curves for inclusion.

### Data analysis

The analysis was performed using Stata v15.1 (Stata, College Station, TX, USA) and the R statistical software v4.0.1 (R Computing, Vienna, Austria). The scripts used for the study are provided to allow reproducibility (Supplementary Data S1).

Prediction equations were derived for FEV_1_, FVC and FEV_1_/FVC. Therefore, only these parameters were systemically collected and analysed. We applied descriptive statistics and multiple regression analysis to the data.^13,14^ Using various modelling strategies, specific reference equations were calculated. We also explored generalised additive models (GAMs) as the GLI did in previous studies.^4,9,15^ A comparison of the model estimates, particularly the adjusted R square, was done, which describes the proportion of variation in the outcome explained by the individual model through the model covariates. We validated the newly generated prediction models using multiple methods. Additionally, bootstrap-based regression model estimates have been reported demonstrating the stability of the model coefficients (Supplementary Table S3).^16,17^

We first determined the *Z*-score for the parameters to interpret spirometry results. A *Z*-score is a number which defines how many standard deviations a raw score is above or below the predicted value. The LLN for spirometric indices is represented by a *Z*-score of -1.64 and corresponds to the fifth percentile of the general population. Participants with a *Z*-score of –1.64 or lower for FEV_1_, FVC or FEV_1_/FVC have lung function indices of or below the fifth percentile and, consequently, were diagnosed with an abnormal lung function in spirometry. Individuals with abnormal spirometry were categorised as follows: obstructive: FEV_1_/FVC ratio < –1.645 and FVC *Z*-score ≥ –1.645, restrictive: FVC *Z*-score < –1.645 and *Z*-score of FEV_1_/FVC ratio ≥ –1.645 or mixed lung function impairment: coexistence of obstruction and restriction.^18^

### Ethical approval and consent to participate

This study was approved by the Mbeya Medical Research and Ethics Committee, Mbeya, Tanzania (Ref No. SZEC-2439/R.A/V.1/04), National Health Research Ethics Review Committee (NIMR/HQ/R.8a/Vol.IX/3027), Dar-es-Salaam, Tanzania; and LMU Munich Ethics Committee, Munich, Germany (Ref No. 786–16). All participants provided written informed consent.

## RESULTS

### Characteristics of study participants

A total of 400 adults were approached for participation in the study. After excluding all individuals who did not meet the inclusion criteria, 393 participants were enrolled and performed spirometry. After applying the ATS/ERS acceptability and repeatability criteria, 343 individuals with usable spirometry curves entered this analysis. All following statistics and equations refer to those 343 participants, who comprised 189 males and 154 females. The anthropometric and spirometric characteristics of the participants are displayed in Table 1. The median age of participants was 32.7 years (interquartile range 18–71.5). Eight participants self-reported to be HIV-positive (3.4%). Overweight and obesity were observed in respectively 33.1% and 27.3% of female participants.

**Table 1.** Anthropometric, demographic and spirometric characteristics of all the participants.

Characteristics	Male (*n* = 189) *n* (%)	Female (*n* = 154) *n* (%)	Total (*n* = 343) *n* (%)
Age, years, mean ± SD	31.20 ± 11.13	34.42 ± 13.04	32.65 ± 12.11
Height, m, mean ± SD	1.68 ± 0.07	1.57 ± 0.06	1.63 ± 0.08
Weight, kg, mean ± SD	68.38 ± 11.94	67.27 ± 14.72	67.88 ± 13.25
BMI, kg/m^2^, mean ± SD	24.32 ± 3.79	27.29 ± 5.54	25.65 ± 4.88
Age group, years			
<20	23 (12.2%)	9 (5.8%)	32 (9.3%)
20–29	82 (43.4%)	67 (43.5%)	149 (43.4%)
30–39	46 (24.3%)	29 (18.8%)	75 (21.9%)
≥40	38 (20.1%)	49 (31.8%)	87 (25.4%)
BMI class			
Underweight	5 (2.7%)	1 (0.7%)	6 (1.8%)
Normal	114 (60.3%)	60 (39.0%)	173 (50.4%)
Overweight	54 (28.6%)	51 (33.1%)	106 (30.9%)
Obese	16 (8.5%)	42 (27.3%)	58 (16.9%)
Marital status			
Single	98 (51.9%)	53 (34.4%)	151 (44.0%)
Married	81 (42.9%)	75 (48.7%)	156 (45.5%)
Living with spouse/partner	3 (1.6%)	1 (0.7%)	4 (1.2%)
Widowed	2 (1.1%)	16 (10.4%)	18 (5.3%)
Divorced	5 (2.7%)	8 (5.2%)	13 (3.8%)
Not known	-	1 (0.7%)	1 (0.3%)
Education			
No formal education	1 (0.5%)	5 (3.3%)	6 (1.8%)
Primary school completed	81 (42.9%)	75 (48.7%)	156 (45.5%)
Secondary school completed	64 (33.9%)	37 (24.0%)	101 (29.5%)
Vocational/college	17 (9.0%)	22 (14.3%)	39 (11.4%)
University or higher	26 (13.8%)	15 (9.7%)	41 (12.0%)
HIV status (self-reported) (*n* = 233, missing observations = 110)			
Negative	100 (94.3%)	125 (98.4%)	225 (96.6%)
Positive	6 (5.7%)	2 (1.6%)	8 (3.4%)
Worked in mines			
No	172 (91.0%)	151 (98.1%)	323 (94.2%)
Yes	17 (9.0%)	3 (2.0%)	20 (5.8%)
Spirometric parameters, mean ± SD			
FVC, L	4.03 ± 0.65	2.90 ± 0.58	3.52 ± 0.83
FVC (% of predicted GLI category: Other)	93.80 ± 11.98	92.85 ± 15.44	93.37 ± 13.63
FVC (% of predicted Knudsen et al. 2011)	105.46 ± 14.12	119.11 ± 21.30	111.59 ± 18.95
FEV_1_, L	3.40 ± 0.60	2.40 ± 0.55	2.95 ± 0.76
FEV_1_ (% of predicted GLI category: Other)	93.61 ± 12.78	89.38 ± 15.97	91.71 ± 14.43
FEV_1_ (% of predicted Knudsen et al. 2011)	110.02 ± 15.76	109.15 ± 19.23	109.63 ± 17.38
FEV_1_/FVC ratio	0.84 ± 0.07	0.82 ± 0.08	0.83 ± 0.07

SD = standard deviation; BMI = body mass index; FVC = forced vital capacity; GLI = Global Lung Function Initiative; FEV_1_ = forced expiratory volume in 1 sec.

### Tanzanian spirometric reference equations

Based on the available spirometry data, we modelled the regression equations for predicting FVC, FEV_1_ and FEV_1_/FVC-ratio (Table 2). Age, height and sex were covariates for predicting FVC and FEV_1_. The Figure shows a positive correlation of FEV_1_/FVC with height and a negative correlation of FEV_1_/FVC with increasing age. We could further demonstrate that FVC and FEV_1_ were highly correlated with each other (Supplementary Figure S1). The ratio for FEV_1_/FVC decreased with age but was not dependent on sex. Regarding stability, the bootstrapped confidence intervals from our data aligned with the estimated regression coefficients estimated by multiple linear regression methods (Supplementary Table S3). We were also exploring more complex models like GAMs with various parameters for their suitability to predict the different parameters. However, they only marginally improved the adjusted R-squared values and were not used to assess lung impairment in our study (Supplementary Table S2). Prediction values generated using different statistical methods also showed robustness in the results (Supplementary Tables S3, S4 and S5, Supplementary Figure S4).

**Table 2.** Newly derived Tanzanian spirometric reference equations.

Outcome (sex-specific)	Tanzanian population
FVC (males)	–4.553 – 0.011*age + 0.053*height RSS = 0.49; adj *R*^2^ = 0.66
FVC (females)	–0.487 – 0.024*age + 0.027*height RSS = 0.49; adj *R*^2^ = 0.66
FEV_1_ (males)	–3.127 – 0.024*age + 0.044*height RSS = 0.42; adj *R*^2^ = 0.70
FEV_1_ (females)	–0.676 – 0.024*age + 0.025*height RSS = 0.42; adj *R*^2^ = 0.70
Ratio FEV_1_/FVC (males)	0.947 – 0.004* age RSS = 0.06; adj *R*^2^ = 0.34
Ratio FEV_1_/FVC (females)	0.947 – 0.004*age RSS = 0.06; adj *R*^2^ = 0.34

FVC = forced vital capacity; adj = adjusted; RSS = residual sum of squares; FEV_1_ = forced expiratory volume in 1 sec.

**Figure.**
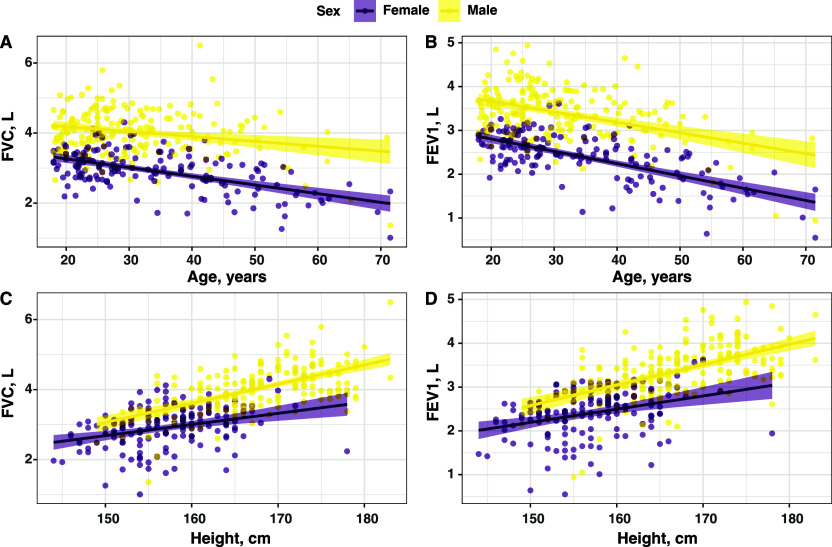
Correlation of FEV_1_ and FVC values (in litres) with height (in cm) and age (in years) according to sex. FEV_1_ = forced expiratory volume in 1 sec; FVC = forced vital capacity.

### Lung impairment in the Tanzanian sample

Using the generated prediction equations, we defined the LLN and the *Z*-scores of the measured values for FEV_1_, FVC and FEV_1_/FVC-ratio. We assessed for impairment, type, and severity based on the acquired Z-scores and LLN values (Tables 3 and 4). Out of 343 participants, a total of 33 participants (Poisson distribution rate 9.6%, 95% confidence interval [CI] 6.6–13.5) had any lung impairment. Seventeen participants had an FVC value below the LLN (= restriction), 12 participants had an FEV_1_/FVC-ratio below the LLN (= obstruction), and 4 participants had both FVC and FEV_1_/FVC-ratio below LLN (= mixed lung function impairment). Regarding severity grading, six participants were classified as having moderate and severe impairment, respectively, and 27 participants had only mild lung function impairment.

**Table 3.** Numbers of participants with different spirometry outcomes (normal, obstruction, restriction or mixed) and severity grades (normal, mild, moderate or severe impairment) based on the Tanzanian reference equations compared to GLI 2012, GLI 2022 and Knudsen et al. 2011^19^ equations. Obstruction severity grading was classified as follows: 1) mild impairment: *Z*-score ≥−2; 2) moderate: *Z*-score <−2 and ≥−2.5; 3) severe: *Z*-score <−2.5. Restriction severity grading was classified as follows: 1) mild impairment: FVC ≥85% LLN FVC; 2) moderate: FVC 55–85% of LLN FVC; 3) severe: FVC <55% of LLN FVC. Using the GLI 2012 and GLI 2022 equations, fewer participants were classified with normal lung function. However, the Knudsen et al.^19^ equations overestimate participants with a normal lung function. The number of participants with moderate and severe lung impairment was increased using the GLI 2012 and 2022 data but was decreased using the Knudsen et al. equations.^19^ GLI = Global Lung Initiative; TZ = Tanzania; FVC = forced vital capacity; LLN = lower limit of normal.

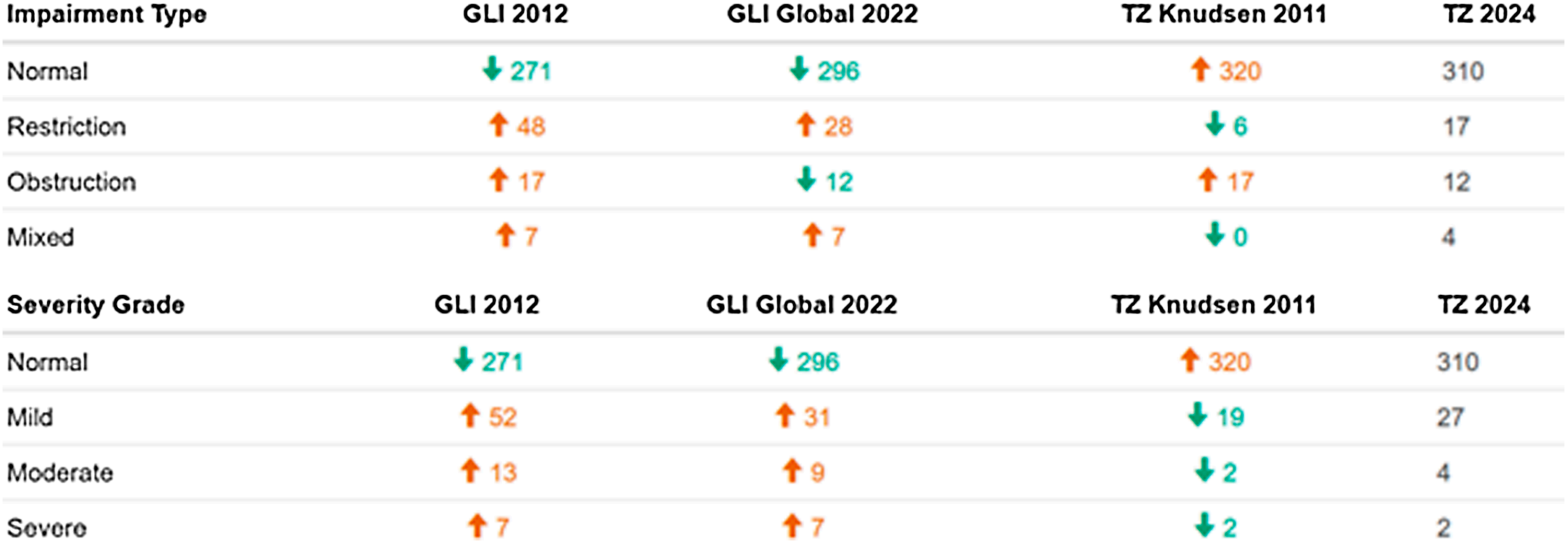

**Table 4.** Comparison of lung function impairment categories: Knudsen et al. equations,^19^ GLI 2012, GLI 2022 and the 2024 Tanzanian reference equations.*

Impairment type and severity grading	Tanzania 2024	Knudsen et al. 2011	GLI 2012	GLI 2022
Normal	310	320	271	296
Obstruction–mild	12	14	13	9
Obstruction–moderate	0	1	4	3
Obstruction–severe	0	2	0	0
Restriction–mild	15	5	39	22
Restriction–moderate	2	1	9	6
Restriction–severe	0	0	0	0
Mixed–mild	0	0	0	0
Mixed–moderate	2	0	0	0
Mixed–severe	2	0	7	7

* While only 23 subjects had abnormal lung function according to the Knudsen et al. 2011^19^ reference standard, 72 subjects had a lung impairment in spirometry if GLI 2012 and standards were used and 47 subjects if GLI 2022 standards were used. However, the 2024 Tanzanian prediction equations classified 33 participants with abnormal lung function.

GLI = Global Lung Initiative.

### Comparison with other reference standards

We compared the outcomes of our participants using different reference standards, including the GLI 2012 race-based, the GLI 2022 race-neutral equations and Knudsen et al.^19^ FEV_1_ and FVC *Z*-scores showed differences when using the different equations. Compared to our newly created reference, a shift towards lower *Z*-scores for FEV_1_ and FVC can be observed when GLI 2012 and 2022 references, respectively, were applied. Contrary to this, a clear change to higher *Z*-scores was observed using the Knudsen et al.^19^ data. The differences in *Z*-scores for FVC and FEV_1_ between our new equations and the existing predictions was about half a standard deviation (Supplementary Figure S2). While our new prediction equations classified 13 participants with a restrictive lung function impairment and four participants with a mixed impairment, the Knudsen et al.^19^ standard would result in only six participants with restrictive and none with mixed impairment whereas the GLI 2012 standards would lead to 48 restriction and seven mixed cases and the GLI 2022 bring about 28 restriction and seven mixed cases.

Analysing obstruction impairment, one can see a higher number of subjects with abnormal FEV_1_/FVC-ratio when using GLI 2012 or Knudsen et al. data:^19^ 12 obstruction cases according to our new reference standard and the GLI 2022 standard versus 17 obstruction cases based on the Tanzanian standard by Knudsen et al.^19^ as well as GLI 2012 (Tables 3 and 4). Not only were the numbers of participants classified as having abnormal lung function different across the four standards, but the number of subjects diagnosed with moderate and severe impairment was also different. Compared to our new data, the use of GLI 2012 and 2022 equations increased, while the Knudsen et al.^19^ references resulted in a reduction of participants with moderate/severe lung impairment (Tables 3 and 4).

## DISCUSSION

To our knowledge, this is the first study in a sufficient number of participants that generated prediction equations for spirometry parameters for a Tanzanian population. Our data supports the heterogeneity within local spirometric reference equations across Africa, as previously shown by Masekela et al.^5^

Many factors may influence lung function in a specific population and, thus, the reference values for spirometric parameters. Previous studies have shown that the most important factors influencing lung function are sex, height, age, and ethnicity/race.^20–23^ The use of race-neutral reference equations is discussed in the literature, as data suggest that race-specific reference standards may lead to systematic misdiagnoses and underestimation of lung function in minorities.^24,25^ Furthermore, data indicates no biological justification for the assumption of different lung functions in individuals of various ethnic backgrounds living in the same region.^26^ However, to discuss using a race-neutral reference standard, it is important to know the differences between ‘normal’ lung function estimates of populations considered ‘healthy’.

This is the first spirometric reference equations study conducted in Southwestern Tanzania. Currently, available references, i.e., GLI 2012/2022 and Knudsen et al.,^19^ did not fit well with our study population in Southwest Tanzania and resulted in an over- and underestimation of patients with relevant lung impairment, respectively. Our data performed better in regard to the newly built models than the previous Tanzanian equations or either GLI equations (Supplementary Table S5). The reasons might be that the Tanzanian study had a smaller sample size and didn´t meet the required standards.^9^ The deviation from the race-neutral equations and the GLI 2012 standard might be that the data set used to generate the two standards lacks data from large proportions of the global population, including sub-Saharan Africa.^27^

Compared to GLI 2012 and 2022, our new Tanzanian formulas generated smaller predicted estimates and higher (more normal) *Z*-scores. Compared to the Knudsen et al. equations,^19^ our new 2024 equations generated higher predicted values and lower (less normal) *Z*-scores for measured values of study participants. Our findings underline the importance of our newly generated data for interpreting spirometry data in Tanzania and East Africa. We demonstrated that the relatively large difference of more than half a standard deviation between estimates of our new standard versus the three available standards could lead to a misclassification of the respiratory health status of individuals, depending on the used equation. The consequences of inappropriate spirometric indices can be immense. The *Z*-scores provided by this study have the advantage of being free of bias related to ethnicity, sex, age and height since *Z*-scores circumvent those biases.^18^ However, it cannot be dismissed that lung damage is prevalent in a certain percentage of our clinically asymptomatic participants. This might be due to a prevalence of risk factors for lung diseases, i.e., air pollution or previous respiratory infections.^28,29^ Whether race-neutral reference equations or ethnicity-adapted equations will be used in the future, the current discussion highlights the importance of the context in which spirometry is performed (i.e., medical or occupational) and the underlying indication (clinical or research) when choosing the reference.

### Limitations

This study has several limitations. First, our study recruited participants from the Mbeya and Songwe regions only, which may limit the applicability. Furthermore, 57 out of 400 participants (14.3%) did not have valid spirometry results. The reason was either the inability to pass validity criteria or due to contraindications. There were no statistically significant differences between the included and non-included subjects for assessed clinical risk factors, including sex, age, height and weight. Nevertheless, other risk factors might have been differently distributed among the groups and, therefore, could have introduced bias, i.e., some statistically significant differences in education level between the two groups (Supplementary Table S1). Additionally, 60.3% of female participants were categorised as either overweight or obese. Studies have demonstrated obese individuals show reduced lung volumes when compared to normal-weight individuals.^30^ However, the obesity rates in our findings are in line with other urban areas of Tanzania.^31^ Furthermore, even though there are more obese females than males in our data, there is no significant increase in impairment in the women´s category (Supplementary Figure S5). Nevertheless, this is one of the major limitations of our study since obesity leads to a reduction in FVC and FEV_1_, therefore suggesting the presence of restrictive respiratory patterns associated with obesity.^32^

## CONCLUSION

This study provides pulmonary function equations in non-smoking Tanzanian adults. The foundation was based on ATS/ERS recommendations. The outcome highlights the need for high-quality, prospectively collected lung function data across Africa. To our knowledge, this is the first Tanzanian study to generate *Z*-scores and LLN values for spirometry testing. The data supports the presumption that GLI 2012 and GLI 2022 may not generally be appropriate for Tanzanians. Hence, the study results will be an important contribution to future analysis of spirometry results from Tanzanian and East African patients with lung disease.

## Supplementary Material


