# Supplementary material for: Spirometric reference equations and lung function testing in adults from Southwestern Tanzania
Source: IJTLD Open. 2024 Oct 1;1(10):473–9. doi: 10.5588/ijtldopen.24.0339 (PMC11467849; doi:10.5588/ijtldopen.24.0339)
Supplement: Supplementary file 1 [file ijtldopen24-0339_Supplementarydata1.pdf]

http://dx.doi.org/10.5588/ijtldopen.24.0339

SUPPLEMENTARY DATA

Spirometric reference equations and lung function testing in adults from Southwestern Tanzania

Supplementary Figure S1 Correlation between observed spirometry outcomes (FEV1 + FVC) according to sex.

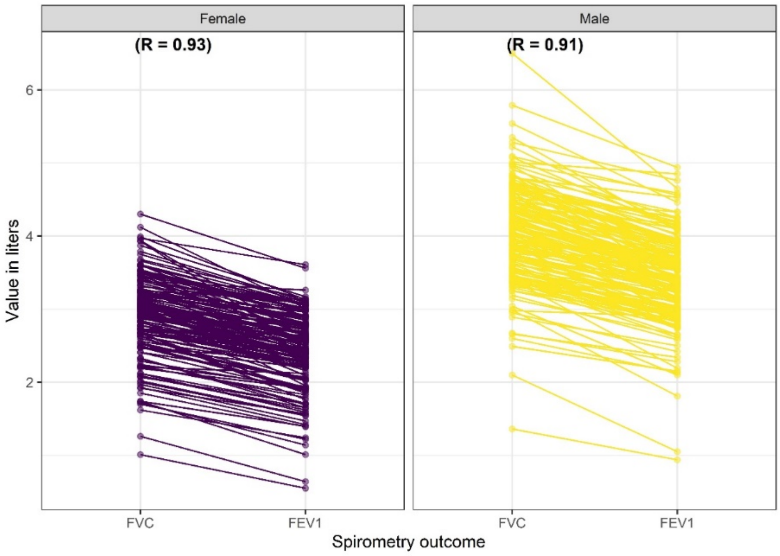

Legend: There is a strong correlation between the values for FVC and FEV1 in both sexes.

Supplementary Figure S2 Distribution of z-scores of study participants for FEV1, FVC and FEV1/FVC-ratio based on the different reference standards.

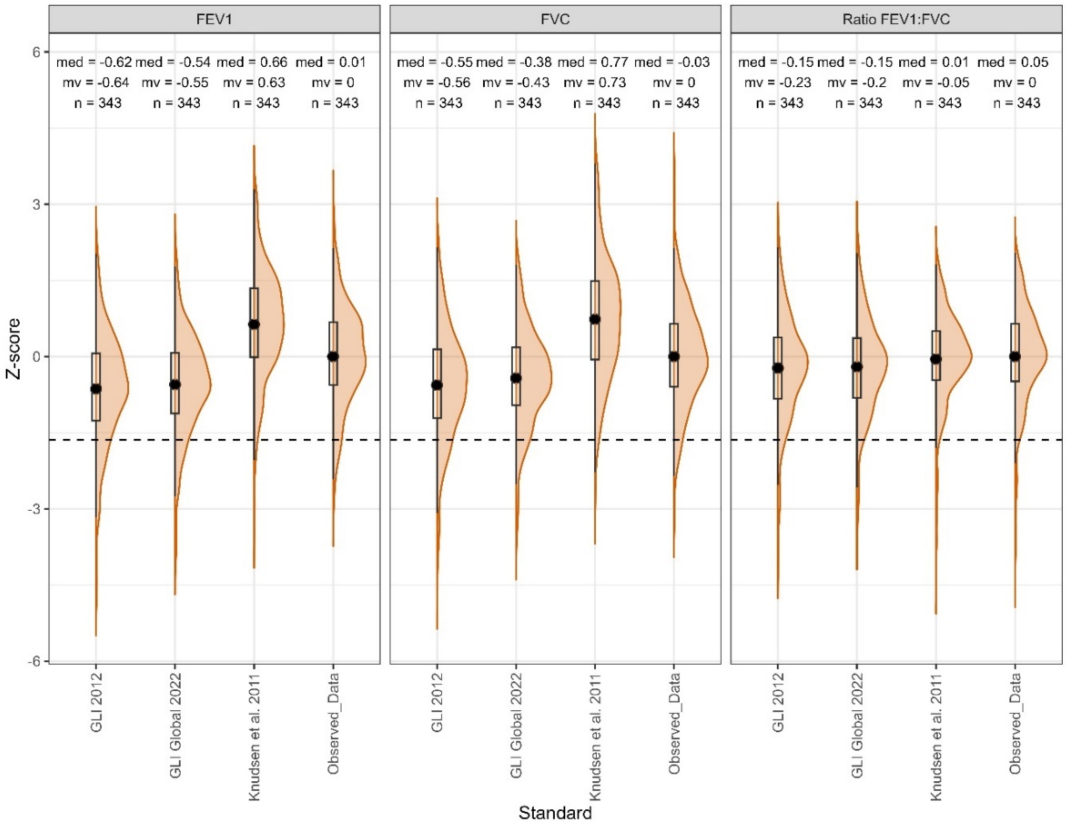

http://dx.doi.org/10.5588/ijtldopen.24.0339

Legend: The distributions are overlapping however the clear shift is seen in the mean value (mv)/median (med) z scores for the fits. Lower z scores and hence more impairment is detected by the GLI 2012 and 2022 standard, as opposed to the fit per Knudsen et al. 2011[19] model for FEV1 and FVC. For the ratio of FEV1: FVC which numerically influences presence of obstruction impairment, very small differences are observed across different model fits.

Supplementary Figure S3 Numbers of participants with different spirometry outcomes (normal, obstruction, restriction or mixed impairment) and severity grades (normal, mild, moderate or severe impairment), based on the Tanzanian prediction equations compared to GLI 2012, GLI 2022 and TZ Knudsen et al. 2011[19] equations.

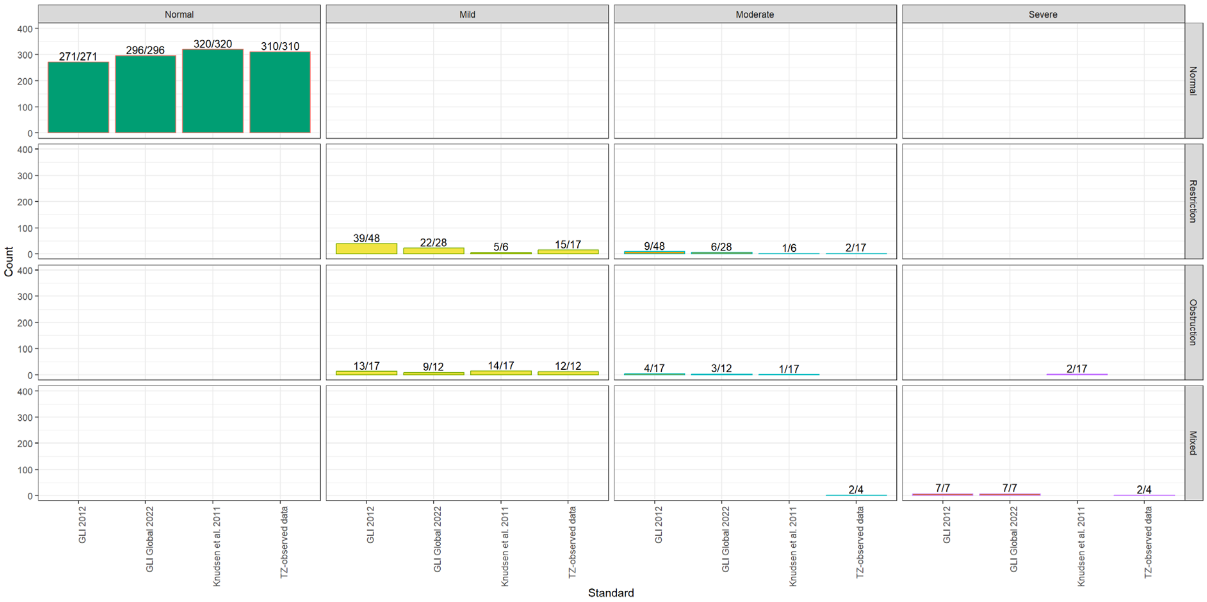

Legend: A lower number of participants was classified as having normal lung function if GLI 2012 and GLI 2022 and a higher number of participants was classified as having normal lung function if the previous Tanzanian equations were used. Also, the number of participants with moderate and severe lung impairment was increased when GLI 2012 or GLI 2022 and decreased when Knudsen et al. 2011[19] reference values were applied.

Supplementary Figure S4 Comparison of observed results of Tanzania 2024 equations modeled in generalized additive models and linear models vs predicted results of Knudsen et al. 2011[19] equations, GLI 2012 and GLI 2022 equations.

http://dx.doi.org/10.5588/ijtldopen.24.0339

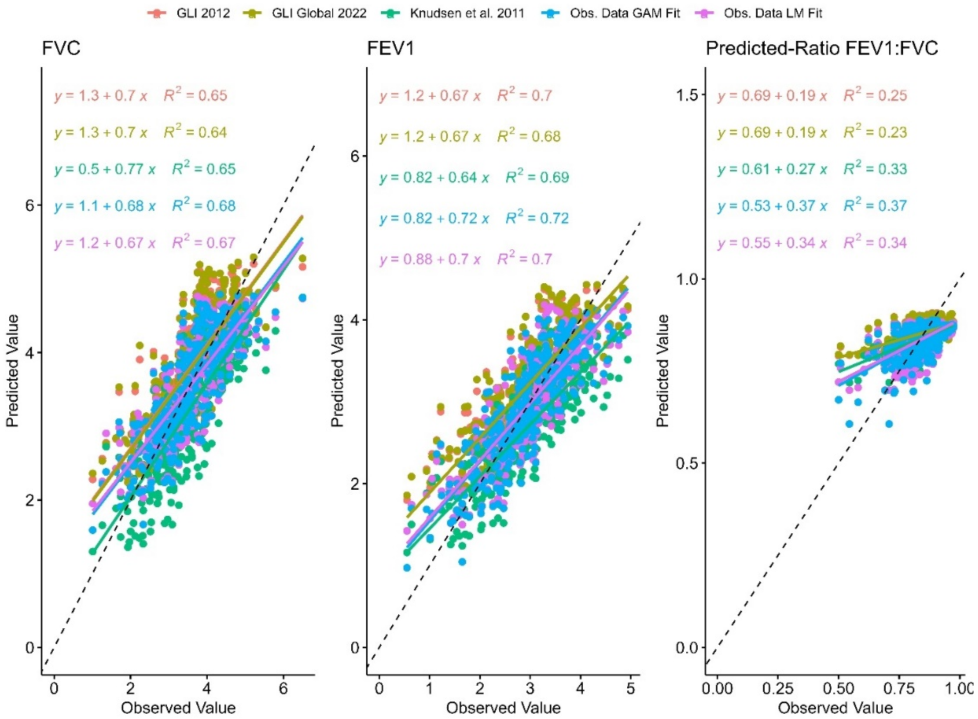

Legend: The fit across the different models are quite similar, and the use of GAM models do not improve the prediction significantly.

Supplementary Figure S5 Correlation of BMI to observed FVC/FEV1, to predicted FVC/FEV1 and to the z-scores of FVC/FEV1

http://dx.doi.org/10.5588/ijtldopen.24.0339

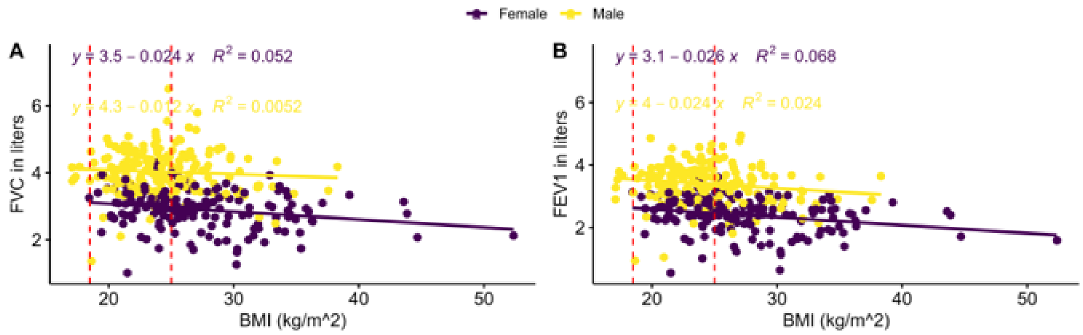

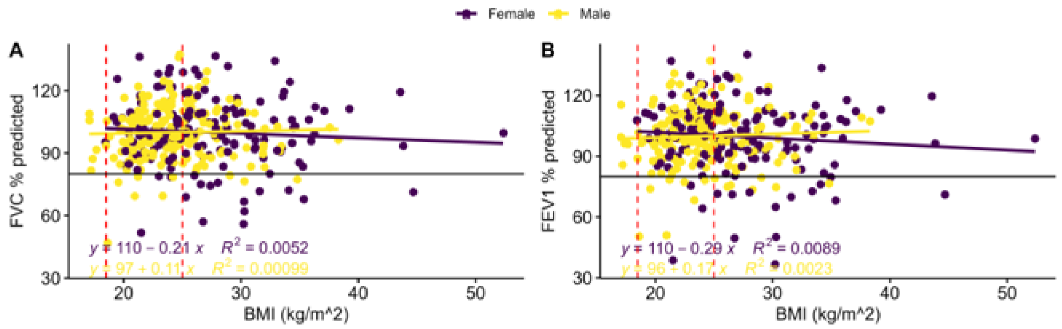

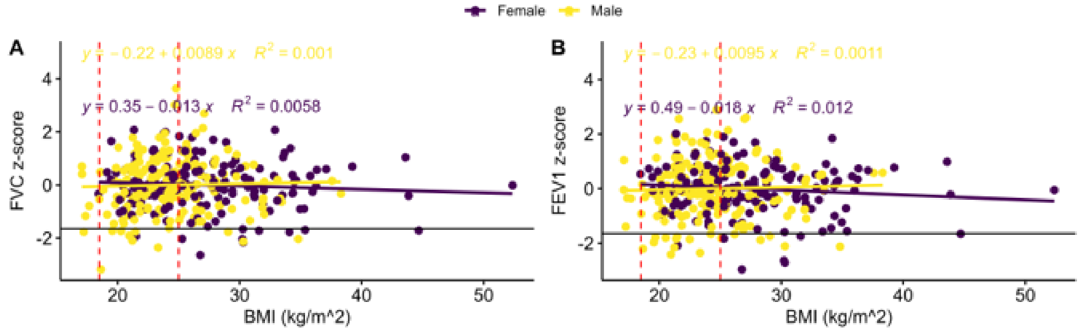

Legend: Looking at the proportion of men compared to women who are below 80% of predicted values, or a z-score < - 1.645, shows that overall lung function is more impaired in men than in women. Of course, there are more women in the obese group than men, simply because there are more obese women in the sample.
So although we have more obese women, the impairment is not significantly higher in women.

Supplementary Table S1 Discussion supporting mostly (see below) no significant differences in clinical parameters for participants with valid spirometry versus invalid spirometry for valid vs not-valid participants.

	Not Valid (N=50)	Valid (N=343)	Total (N=393)	p value
**Sex (M/F)**				0.346
Female	26 (52.0%)	154 (44.9%)	180 (45.8%)	
Male	24 (48.0%)	189 (55.1%)	213 (54.2%)	
**Age (years)**				0.090
Mean (SD)	35.910 (16.049)	32.647 (12.115)	33.062 (12.705)	
Range	18.500 - 82.500	18.000 - 71.500	18.000 - 82.500	
**Agegroup**				0.713
Under 20	3 (6.0%)	32 (9.3%)	35 (8.9%)	
Between 20-30	21 (42.0%)	149 (43.4%)	170 (43.3%)	
Between 30-40	10 (20.0%)	75 (21.9%)	85 (21.6%)	
Over 40	16 (32.0%)	87 (25.4%)	103 (26.2%)	
**Weight (kg)**				0.746

http://dx.doi.org/10.5588/ijtldopen.24.0339

Mean (SD)	67.222 (15.086)	67.883 (13.250)	67.799 (13.478)	
Range	45.000 - 127.000	42.000 - 121.000	42.000 - 127.000	
**Height (cms)**				0.636
Mean (SD)	162.160 (8.853)	162.773 (8.495)	162.695 (8.532)	
Range	145.000 - 185.000	144.000 - 183.000	144.000 - 185.000	
**BMI (kg/m_sq)**				0.840
Mean (SD)	25.810 (6.551)	25.654 (4.882)	25.674 (5.115)	
Range	16.563 - 46.088	17.078 - 52.372	16.563 - 52.372	
**Education**				0.016
No formal education	3 (6.0%)	6 (1.7%)	9 (2.3%)	
Primary completed	14 (28.0%)	156 (45.5%)	170 (43.3%)	
Secondary completed	15 (30.0%)	101 (29.4%)	116 (29.5%)	
Vocational/college	5 (10.0%)	39 (11.4%)	44 (11.2%)	
University	13 (26.0%)	39 (11.4%)	52 (13.2%)	
Higher than University	0 (0.0%)	2 (0.6%)	2 (0.5%)	
**Mine work (Y/N)**				0.962
No	47 (94.0%)	323 (94.2%)	370 (94.1%)	
Yes	3 (6.0%)	20 (5.8%)	23 (5.9%)	
**Cultural Practises(Y/N)**				0.132
N-Miss	1	1	2	
No	37 (75.5%)	221 (64.6%)	258 (66.0%)	
Yes	12 (24.5%)	121 (35.4%)	133 (34.0%)	
**Cooking method(primary)**				0.553
Electricity	1 (2.0%)	8 (2.3%)	9 (2.3%)	
Gas	21 (42.0%)	113 (32.9%)	134 (34.1%)	
Charcoal	14 (28.0%)	135 (39.4%)	149 (37.9%)	
Wood	14 (28.0%)	85 (24.8%)	99 (25.2%)	
Others	0 (0.0%)	2 (0.6%)	2 (0.5%)	

Legend: There are some differences in education levels. Those are significant but mainly due to small numbers.

Supplementary Table S2 Regression results of the Tanzanian model comparing the estimates of a multiple linear regression model and a GAM model

	Dependent variable					
	FVC		FEV1		Ratio FEV1:FVC	
	OLS	GAM (continuous)	OLS	GAM (continuous)	OLS	GAM (continuous)
SexMale	0.595*** (0.459, 0.731)	-3.530*** (-6.179, -0.881)	0.522*** (0.406, 0.638)	-2.359** (-4.632, -0.086)		0.010 (-0.002, 0.023)
Age	-0.018*** (-0.022, -0.013)		-0.024*** (-0.028, -0.021)		-0.003*** (-0.004, -0.003)	
poly(Age, 5)1		-5.109*** (-6.433, -3.785)		-5.960*** (-7.096, -4.824)		-0.772*** (-0.934, -0.610)
poly(Age, 5)2		-1.552** (-2.938, -0.166)		-0.930 (-2.120, 0.259)		-0.031 (-0.200, 0.138)
poly(Age, 5)3		0.683 (-0.713, 2.080)		0.273 (-0.925, 1.471)		-0.160* (-0.330, 0.011)
poly(Age, 5)4		-0.445 (-1.804, 0.914)		-0.575 (-1.741, 0.591)		-0.120 (-0.285, 0.046)
poly(Age, 5)5		-0.462 (-1.814, 0.889)		-0.542 (-1.702, 0.618)		-0.091 (-0.256, 0.075)

Height	0.044*** (0.036, 0.052)	0.025*** (0.012, 0.038)	0.037*** (0.030, 0.044)	0.024*** (0.013, 0.035)		
SexMale:poly(Age, 5)1		2.362** (0.432, 4.292)		0.871 (-0.785, 2.528)		-0.035 (-0.271, 0.200)
SexMale:poly(Age, 5)2		-0.218 (-2.162, 1.727)		-0.575 (-2.243, 1.094)		-0.112 (-0.347, 0.123)
SexMale:poly(Age, 5)3		-1.045 (-2.982, 0.892)		-0.070 (-1.732, 1.593)		0.226* (-0.011, 0.463)
SexMale:poly(Age, 5)4		-0.364 (-2.290, 1.561)		-0.166 (-1.819, 1.486)		0.144 (-0.091, 0.379)
SexMale:poly(Age, 5)5		1.974** (0.083, 3.864)		2.112** (0.490, 3.734)		0.202* (-0.029, 0.434)
SexMale:Heig ht		0.026*** (0.009, 0.042)		0.018** (0.004, 0.032)		
Constant	0.947*** (-4.633, -2.071)	-0.993 (-3.061, 1.075)	-2.550*** (-3.639, -1.460)	-1.283 (-3.058, 0.491)	-3.352*** (0.929, 0.965)	0.827*** (0.818, 0.837)
AIC	499	482.9	388.1	378	-962.3	-959
Observations	343	343	343	343	343	343
R2	0.649		0.695		0.337	
Adjusted R2	0.645	0.671	0.693	0.710	0.335	0.348
Log Likelihood		-241.437		-188.984		479.502
UBRE		0.238		0.176		0.004
Residual Std. Error	0.496 (df = 339)		0.422 (df = 339)		0.059 (df = 341)	
F Statistic	208.537*** (df = 3; 339)		257.751*** (df = 3; 339)		173.458*** (df = 1; 341)	
Note:						*p<0.1; **p<0.05; ***p<0.01

Legend: The GAM models do not improve significantly compared to the linear regression models with multiple parameters.

Supplementary Table S3 Bootstrap estimates of the regression analysis

	FVC			FEV1			Ratio FEV1:FVC		
	Beta	95% CI		Beta	95% CI		Beta	95% CI	
(Intercept)	-0.4872	-2.5167	1.6784	-0.4993	-2.2686	1.3038	0.9393	0.9167	0.9642
SexMale	-4.0660	-7.0830	-1.3350	-2.7820	-5.1270	-0.4970	0.0102	-0.0029	0.0231
Age	-0.0237	-0.0294	-0.0181	-0.0270	-0.0320	-0.0225	-0.0034	-0.0042	-0.0028
Height	0.0268	0.0133	0.0396	0.0244	0.0130	0.0357			
SexMale: Age	0.0125	0.0021	0.0219	0.0055	-0.0032	0.0136			
SexMale: Height	0.0265	0.0095	0.0448	0.0194	0.0052	0.0341			
R2	0.6651	0.6092	0.7061	0.7030	0.6514	0.7418	0.3420	0.2551	0.4260
Considering important covariates of interest only									
	FVC			FEV1			Ratio FEV1:FVC		
	Beta	95% CI		Beta	95% CI		Beta	95% CI	
(Intercept)	-0.4872	-2.5167	1.6784	-0.6757	-2.4757	1.0528	0.9467	0.9277	0.9686
SexMale	-4.0660	-7.0830	-1.3350	-2.4517	-4.8500	-0.1580			
Age	-0.0237	-0.0294	-0.0181	-0.0244	-0.0286	-0.0204	-0.0035	-0.0042	-0.0029

Height	0.0268	0.0133	0.0396	0.0250	0.0140	0.0364			
SexMale: Age	0.0125	0.0021	0.0219						
SexMale: Height	0.0265	0.0095	0.0448	0.0185	0.0042	0.0334			
R2	0.6651	0.6074	0.7063	0.7011	0.6507	0.7399	0.3420	0.2566	0.4275

Legend: Stability of the regression equations was evaluated by comparing regression estimate to the non-parametric bootstrap estimates. The corresponding 95% confidence intervals were then based on 10000 resamples from the collected data.

Supplementary Table S4 Linear regression estimates for the observed data with the corresponding 95% confidence intervals.

	**FVC**			**FEV 1**			**rfev 1.fvc**		
	Estimates	*CI*	*p*	*Estimates*	*CI*	*p*	*Estimates*	*CI*	*p*
Predictors	-0.4872	-2.5871 – 1.6127	0.648	-0.6757	- 2.4692 – 1.1177	0.459	0.9467	0.9286 – 0.9648	**<0.001**
(Intercept)	-4.066	-6.7511 – -1.3809	**0.003**	-2.4517	-4.7214 – -0.1820	**0.034**			
Sex [Male]	-0.0237	-0.0296 – -0.0177	**<0.001**	-0.0244	-0.0281 – -0.0207	**<0.001**	-0.0035	-0.0040 – -0.0030	**<0.001**
Age	0.0268	0.0136 – 0.0400	**<0.001**	0.025	0.0136 – 0.0363	**<0.001**			
Height	0.0125	0.0038 – 0.0212	**0.005**						

Supplementary Table S5 Sensitivity analysis of the prediction equations based on the observed Tanzanian data.

**Outcome**	**Training 2/3; Testing 1/3 – 1 Random Draw**	**Leave One Out Cross Validation - LOOCV**	**10-Fold Cross-Validation**	**5 Repeated 10-Fold Cross-Validation**	**GLI 2012**	**GLI 2022**	**TZ 2011 published equations**
FVC	Rsquared -0.69	Rsquared -0.65	Rsquared -0.66	Rsquared -0.66	Rsquared -0.65	Rsquared -0.64	Rsquared -0.65
	RMSE -0.49	RMSE -0.49	RMSE -0.49	RMSE -0.49	RMSE -0.55	RMSE -0.56	RMSE -0.60
	MAE -0.39	MAE -0.38	MAE -0.38	MAE -0.38	MAE -0.43	MAE -0.43	MAE -0.48
FEV1	Rsquared -0.72	Rsquared -0.69	Rsquared -0.70	Rsquared -0.71	Rsquared -0.70	Rsquared -0.68	Rsquared -0.69
	RMSE -0.42	RMSE -0.42	RMSE -0.42	RMSE -0.42	RMSE -0.49	RMSE -0.49	RMSE -0.50
	MAE -0.34	MAE -0.33	MAE -0.33	MAE -0.33	MAE -0.38	MAE -0.38	MAE -0.40
Ratio FEV1: FVC	Rsquared -0.35	Rsquared -0.33	Rsquared -0.34	Rsquared -0.35	Rsquared -0.25	Rsquared -0.23	Rsquared -0.33
	RMSE -0.05	RMSE -0.06	RMSE -0.06	RMSE -0.06	RMSE -0.07	RMSE -0.07	RMSE -0.06

http://dx.doi.org/10.5588/ijtldopen.24.0339

	MAE -0.04	MAE -0.04	MAE -0.04	MAE -0.04	MAE -0.05	MAE -0.05	MAE -0.04

Legend: The data was divided into two parts (2/3 and 1/3 of the data), where one part (2/3 of the data) was used to train the model and the second part (1/3 of the data) was used to test the model based on the trained data. This would allow for a snapshot of one potential instance. Hence, we used several cross-validation (CV) methods such as Jackknife estimator (leave one out CV (LOOCV)), 10fold CV and 10fold CV with 5 repetitions which are based on resampling methods and provide multiple instances. The newly generated prediction models were evaluated for predictive efficacy using the measures of root mean square errors (RMSE), mean absolute error (MAE) and R2 (describing the squared correlation between observed and predicted in the test data set). We see that the predictions performances are consistent across different methods.

Supplementary Data S1 Data Analyses Manuscript Healthy Volunteers Tanzania
file:///C:/Users/Rebekka%20Wenzel/Desktop/AdultspreFinalVersion/BMI%20Graphs/Analysis-Markdown-Document.html

-further information-

A. Performance of spirometry and related quality control procedures:

All study procedures related to spirometry testing, quality control of spirometry results and data analysis were performed according to ATS/ERS guidelines published in 2005[1]. After giving consent, the volunteers were asked to answer an extensive set of questions regarding exclusion criteria as well as any underlying contraindications to perform spirometry. Participants were excluded if they reported any history of pulmonary disease or showed any signs of a past or current pulmonary disease, e.g. a respiratory infection, asthma, COPD or cystic fibrosis. One main focus was on detecting any potential TB symptoms, a past TB treatment or any other indication for a current or past TB diagnosis.

In case the participants screened positive for TB or other respiratory symptoms, they were excluded from the study and referred to the nearest health centre. Furthermore, all volunteers who did screen positive for TB were offered to give a sputum sample immediately in order to diagnose an active case of tuberculosis.

For this study, a list of potential medical contraindications, which could be adversely affected by the maximal pressures in thorax and abdomen as well as by the increase in myocardial demand that are associated with spirometry was generated by the involved investigators, external monitors and experts, as such a list was not included in ARS/ERS guidelines from 2005[1,2].

Contraindications to perform spirometry included a history of cardiovascular disease, muscular disorders, a history of cataract or glaucoma, a history of sickle cell disease, lung surgery, a severe chest trauma or a recent surgery/hospitalization.

Subsequent to answering questions in regard to their medical history, volunteers were asked to answer a detailed socio-demographic questionnaire as well as a Quality-of-Life questionnaire. The following lung function test comprised two parts: measuring anthropometric data as well as the spirometry test itself. Body weight in kg was measured with a portable scale to the closest 0.1 kg while wearing minimal clothing and no shoes. Standing height in m was measured with a portable stadiometer to the closest 0.01m, head in Frankfort plane position, while wearing no shoes.

Spirometry was performed using the ndd EasyOne Air spirometer (ndd Medizintechnik AG) which is able to transform ATP (ambient temperature, pressure) conditions to BTPS (body temperature, pressure, water vapor saturated) conditions. The correction is used to convert flow and volume measured at ambient conditions to the conditions within the lungs. The handheld spirometer works with ultrasound technology without the need for periodic calibration. Nonetheless, the study team performed a weekly calibration check in order to guarantee sound spirometry results. Measurement of spirometric indices was done in sitting position with a nose clip obstructing the nostrils.

http://dx.doi.org/10.5588/ijtldopen.24.0339

The testing procedure was structured according to the following pattern: First, the study participant was given an explanation of the procedure in their local language, then a trained study team member demonstrated the test in front of the participant, followed by a demonstration of the maneuver – without the spirometer – by the participant. Finally, the volunteer performed the spirometry test with the device.

Therefore, the individual was asked to inflate their lungs to maximal capacity before exhaling with maximum speed and effort. The exhalation should then be continued till the point of complete expiration. After completing the spirometry test a trained study team member examined the curve according to ATS/ERS acceptability and repeatability criteria[3]. In case the curve didn´t meet those criteria, the operator made suggestions on how to improve the technique of the participant.

A spirogram is acceptable if there is no hesitation in the beginning, no cough during the first second, no air leak, no evidence of obstructed mouthpiece, no glottic closure as well as fulfils the necessary end of forced expiration (EOFE) criteria (**Supplementary Table S6**). A spirometry test with an unacceptable start or unusable curve was discarded before applying repeatability criteria (**Supplementary Table S7**). If the individual completed 3 acceptable blows which met the repeatability criteria, the session was finished. However, if the acceptability or repeatability criteria were not met, the testing proceeded until the criteria were met or the volunteer completed a total of 8 unacceptable tests. After completing an unsuccessful set of 8, the participant was asked if they wanted to repeat the spirometry test after a lengthy break or if they wanted to finish the pulmonary function test permanently.

Spirometry data were stored electronically using EasyWare software (NDD Medizintechnik AG, Technoparstrasse 1, CH-8005 Zürich, Schweiz) and uploaded onto a server hosted by the study team at Ludwig- Maximilian-University in Munich (LMU Klinikum). Quality control was performed on 100% of produced spirometry curves. Errors of each spirometry attempt were categorized as shown in **Supplementary Table S6** below. Afterwards, each spirometry attempt was quality graded according to **Supplementary Table S7**. After the release of an updated version of ATS/ERS spirometry guidelines in 2019[3], all spirometry attempts with a grading of “2” or below (**Supplementary Table S7**) were individually reviewed again by an expert from PATS (Pan African Thoracic Society) and graded according to the new guidelines[3]. For this manuscript, all spirometry results of at least grade “E” were considered as **valid** (**Supplementary Figure S6)**[3].

Supplementary Table S6: List of potential errors of spirometry curves

Error	Abbre-viation	Definition	Reference/ source
High PEFT	p (PEFT)	PEFT ≥150msecs	BOLD QC requirements, which relaxed the ndd cut off of 120ms
High BEV	b (BEV)	BEV ≥150ml<br>**AND**<br>BEV ≥5% of FVC	ATS criteria, NIOSH, BOLD QC requirements state that for a curve to be included BEV must be <5% or <150ml, whichever is greater.
Non-maximal effort	e (effort)	Marked lack of peak, indicating weak blast<br>**OR**<br>Markedly reduced peak compared to other curves, indicating poor filling of lungs at start of test	BOLD QC requirements<br>ATS criteria<br>NIOSH guidelines
Early termination of expiration	t (termination)	Insufficient expiratory phase on volume-time curve – duration of expiration for < 6 secs OR failure to reach plateau of ≥1 sec<br>**OR**	BOLD QC requirements<br>ATS criteria<br>NIOSH guidelines

		Sharp early drop to 0 on flow-volume curve	
Extra breath	x (extra)	Visible extra breath on flow-volume and or the volume-time curves	BOLD QC requirements<br>NIOSH guidelines
Glottis closure that influences measurement	g (glottis)	Abrupt flat line on volume-time curve, with sharp drop to 0 on flow-volume curve	BOLD QC requirements<br>ATS criteria<br>NIOSH guidelines
Leak	l (leak)	Descent of volume-time curve, after peak is reached, with 'back-track' of flow-volume curve at the end of expiration	BOLD QC requirements<br>ATS criteria<br>NIOSH guidelines
Obstructed mouthpiece	o (obstruction)	Artefact in the flow-volume and volume-time curves, felt to be significant enough to affect measurement	BOLD QC requirements<br>ATS criteria<br>NIOSH guidelines
Cough that affects measurement	c (cough)	Cough within 1st second which is likely to alter FEV1, or a later cough which causes early termination.	BOLD QC requirements<br>ATS criteria<br>NIOSH guidelines
Zero flow error	z (zero)	Continuous rise of volume-time curve, with no plateau, and long tail on flow-volume curve, which is felt related to error rather than obstructive impairment	BOLD QC requirements<br>NIOSH guidelines

Supplementary Table S7: Quality grading of usable curves, including repeatability assessment

Validity classification for FEV1 and FVC	Abbre-viation	Definition if FEV or FV volume ≥1 L	Difference in definition, if FEV or FVC volume <1L
Unable to assess	0	< 2 usable curves, so unable to compare	No change
Not valid	1	≥2 usable curves<br>**AND**<br>Difference between highest usable and second highest usable FEV_1 >250ml<br>Difference between highest usable FVC and second highest usable FVC >250ml	Difference is >200ml
Borderline	2	≥2 usable curves<br>**AND**<br>Difference between highest usable FEV_1 and second highest usable FEV_1 is >200ml but ≤250ml<br>Difference between highest usable FVC and second highest usable FVC is >200ml but ≤250ml	Difference is >150ml but ≤200ml
**Valid** – BOLD QC criteria	**3**	≥2 usable curves<br>**AND**<br>Difference between highest usable FEV_1 and second highest usable FEV_1 is >150ml but ≤200ml	Difference is >100ml but ≤150ml

		Difference between highest usable FVC and second highest usable FVC >150ml but ≤200ml	
**Valid** – ATS QC criteria	**4**	≥2 usable curves<br>**AND**<br>Difference between highest usable FEV_1 and second highest usable FEV_1 ≤150ml<br>Difference between highest usable FVC and second highest usable FVC ≤150ml	Difference is ≤150ml

Supplementary Figure S6: Grading System for FEV1 and FVC according to new ATS/ERS guidelines

Grade	Number of Measurements	Repeatability: Age >6 yr	Repeatability: Age ≤6 yr*
A	≥3 acceptable	Within 0.150 L	Within 0.100 L*
B	2 acceptable	Within 0.150 L	Within 0.100 L*
C	≥2 acceptable	Within 0.200 L	Within 0.150 L*
D	≥2 acceptable	Within 0.250 L	Within 0.200 L*
E	≥2 acceptable	>0.250 L	>0.200 L*
	OR 1 acceptable	N/A	N/A
U	0 acceptable AND ≥1 usable	N/A	N/A
F	0 acceptable and 0 usable	N/A	N/A

Definition of abbreviation: N/A = not applicable.
The repeatability grade is determined for the set of prebronchodilator maneuvers and the set of post-bronchodilator maneuvers separately. The repeatability criteria are applied to the differences between the two largest FVC values and the two largest FEV_1 values. Grade U indicates that only usable but not acceptable measurements were obtained. *Although some maneuvers may be acceptable or usable at grading levels lower than A, the overriding goal of the operator must be to always achieve the best possible testing quality for each patient.* Adapted from Reference 114.
*Or 10% of the highest value, whichever is greater; applies for age 6 years or younger only.

Legend to Supplementary Figure S6: this figure is identical to Table 10 in Standardization of Spirometry 2019 Update by ATS/ERS[3].

B. List of abbreviations:

ATS	American Thoracic Society
BOLD	Burden of Lung Disease- study
ERS	European Respiratory Society
GLI	Global Lung Initiative
FEV1	Forced Expiratory Volume in 1 second
FVC	Forced Vital Capacity
ndd	NDD Medical Technologies
NIOSH	National Institute for Occupational Safety and Health
PATS	Pan African Thoracic Society
QC	Quality Control

C. References:

1. Miller MR, Hankinson J, Brusasco V, Burgos F, Casaburi R, Coates A, et al. Standardisation of spirometry. Eur Respir J. 2005;26(2):319-38.
2. Miller MR, Crapo R, Hankinson J, Brusasco V, Burgos F, Casaburi R, et al. General considerations for lung function testing. Eur Respir J. 2005;26(1):153-61.
3. Graham BL, Steenbruggen I, Miller MR, Barjaktarevic IZ, Cooper BG, Hall GL, et al. Standardization of Spirometry 2019 Update. An Official American Thoracic Society and European Respiratory Society Technical Statement. Am J Respir Crit Care Med. 2019;200(8):e70-e88.
